# Bioenergetics And Metabolic Patterns in Early Onset Severe Sepsis Or Trauma

**DOI:** 10.1186/2197-425X-3-S1-A43

**Published:** 2015-10-01

**Authors:** T Tavladaki, AM Spanaki, H Dimitriou, AV Kozlov, JC Duvigneau, A Weidinger, E Kondili, D Georgopoulos, G Briassoulis

**Affiliations:** University of Crete/University Hospital, PICU, Heraklion, Greece; University of Crete, Medical School, Paediatric Haematology Oncology, Heraklion, Greece; Ludwig Boltzmann Institute for Experimental and Clinical Traumatology in the AUVA, Vienna, Austria; University of Veterinary Medicine, Vienna, Austria; University of Crete/University Hospital, ICU, Heraklion, Greece

## Introduction

Mitochondrial respiration in vitro is inhibited in severe illness. The mitochondria are one of the major targets for NO.

## Objectives

We examined the first 24-hours differences of ATP, NO_2_¯ and NO_3_¯ in patients with severe sepsis (SS) or trauma-related systemic inflammatory response syndrome (SIRS) compared to healthy-controls (H) and examined their relations to intracellular heat shock proteins (HSP)-72 and -90α, metabolism and outcome.

## Methods

Seventy-eight adults (SS/22; non-infectious SIRS /23; healthy (H)/33) were included in our study. Energy expenditure (EE) of patients was measured with the Gas Module E-COVX. Patients were classified as hypermetabolic, normometabolic, and hypometabolic when the EE were >110%, 90-110% and, < 90% of the predicted basal metabolic rate, respectively. HSPs expressions in monocytes (m) or neutrophils (n) and the levels of amino acids in plasma were determined using flow cytometry and HPLC, respectively. ATP concentrations were measured by the luciferase luminescent assay. NO_2_¯ and NO_3_¯ determination was performed using the Sievers Nitric Oxide Analyzer.

## Results

SS and SIRS patients predominantly manifested hypometabolic (SS 41%, SIRS 65%) or hypermetabolic (SS 41%, SIRS 17.4%) rather than normometabolic pattern (SS 18.2% vs. 17.4%). The hypometabolic pattern was associated with increased mortality (p < 0.01); accompanied by decreased concentrations of arginine, citruline, glutamine (p < 0.04) and mHSP72 and nHSP72 expression (p < 0.03). Patients with SS had lower ATP (184 ± 133 vs. 895 ± 863nM), and NO2¯ (211 ± 56 vs. 280 ± 53nM, p < 0.03) and SIRS patients lower NO3¯ (p < 0.005) and NO2¯ (p < 0.02) compared to controls. Comparison between SS and SIRS showed, that SS had repressed mHSP72 (p < 0.002) and nHSP72 expression (p < 0.01), decreased SID (p < 0.03) and increased NO3¯ levels (p < 0.01). ATP was correlated with glutamine (p < 0.05), NO2¯ with citruline (p < 0.03) and glucine (p < 0.05), and NO3¯ with the metabolic pattern (p < 0.05), CRP (p < 0.02), SAPS3 (p < 0.02) and inversely with pH (p < 0.002).

## Conclusions

Early ons et hypometabolism and bioenergitic failure characterize critical illness and are related to intracellular HSP72 repression, severity of illness and outcome. The patterns of NO metabolites in plasma suggest that the changes in ATP and HSP72 are linked to NO metabolism.

## Grant Acknowledgment

This research has been co-financed by the European Union (European Social Fund (ESF)) and Greek national funds through the Operational Program "Education and Lifelong Learning" of the National Strategic Reference Framework (NSRF)-Research Funding Program: THALES.

